# Severe Suspected Metformin-Associated Lactic Acidosis With Relatively Preserved Serum Creatinine at Presentation: Sequential Intermittent Hemodialysis and Continuous Venovenous Hemodialysis

**DOI:** 10.7759/cureus.116607

**Published:** 2026-09-20

**Authors:** Mahmoud Hassanein, Christina Rodriguez, Krista L Lentine

**Affiliations:** 1 Nephrology, SSM (Sisters of St. Mary) Health Saint Louis University Hospital, St. Louis, USA; 2 Dialysis Services, SSM (Sisters of St. Mary) Health Saint Louis University Hospital, St. Louis, USA; 3 Nephrology, SSM (Sisters of St. Mary) Health Saint Louis University Hospital Transplant Center, St. Louis, USA

**Keywords:** acute kidney injury, continuous veno-venous hemodialysis, extracorporeal treatment, intermittent hemodialysis, metformin-associated lactic acidosis

## Abstract

Metformin-associated lactic acidosis (MALA) is an uncommon but potentially fatal complication of metformin therapy in which extracorporeal treatment may be required for severe acidemia or markedly elevated lactate levels. We report the case of a 47-year-old man with type 2 diabetes mellitus treated with metformin who presented with encephalopathy, profound hypoglycemia, hypothermia, and severe high-anion-gap metabolic acidosis. Lactate was 24.1 mmol/L and peaked at 25.5 mmol/L, while serum total carbon dioxide was <5 mmol/L. Despite the severity of the metabolic disturbance, serum creatinine was 1.18 mg/dL on presentation, although he had experienced recent acute kidney injury. Toxicologic evaluation did not identify an alternative major cause of the acidosis, although urine testing was positive for amphetamine/methamphetamine, suggesting a potentially multifactorial process. The patient received supportive care and repeated intravenous sodium bicarbonate before extracorporeal treatment. Lactate had already declined from 25.5 to 16.0 mmol/L before dialysis began, while acidemia partially improved after bicarbonate administration. High-efficiency intermittent hemodialysis (iHD) was initiated for severe suspected MALA as the rapid initial phase of extracorporeal treatment, followed by continuous venovenous hemodialysis to provide sustained therapy toward biochemical treatment targets; hemodynamic intolerance during iHD further supported the transition. Lactate subsequently normalized without rebound, and kidney function recovered without further renal replacement therapy. This case highlights that severe suspected MALA may warrant extracorporeal treatment despite relatively preserved creatinine at presentation and illustrates the importance of aligning biochemical trends with treatment timing when assessing the contribution of dialysis to lactate and acid-base correction.

## Introduction

Metformin is a first-line therapy for type 2 diabetes mellitus and has a favorable safety profile; however, metformin-associated lactic acidosis (MALA) is a rare but potentially life-threatening complication. MALA is generally considered in a patient receiving metformin who develops high-anion-gap metabolic acidosis with an elevated serum lactate concentration, commonly defined as lactate >5 mmol/L and pH <7.35. Importantly, the term metformin-associated lactic acidosis does not necessarily imply that metformin is the sole cause of the metabolic disturbance. In critically ill patients, metformin accumulation frequently coexists with other causes of increased lactate production or impaired lactate clearance, including shock, sepsis, tissue hypoxia, hepatic dysfunction, and acute kidney injury [1].

The pathogenesis of MALA involves impaired oxidative metabolism and reduced lactate utilization. Metformin alters mitochondrial energy metabolism, and inhibition of mitochondrial respiratory-chain complex I has traditionally been implicated, particularly at high intracellular concentrations, although the precise contribution of this mechanism at therapeutic concentrations remains debated [2]. Metformin also alters cellular redox balance and suppresses hepatic gluconeogenesis from redox-dependent substrates such as lactate, thereby reducing lactate utilization. Increased intestinal glucose utilization and lactate generation may provide an additional source of circulating lactate. Thus, when metformin accumulates, hyperlactatemia may result from a combination of increased production and impaired metabolic clearance [2,3].

Metformin is not significantly metabolized and is eliminated largely unchanged by the kidneys. Clinically significant toxicity therefore commonly develops when a precipitating event reduces renal metformin clearance. Acute kidney injury (AKI) related to dehydration, gastrointestinal losses, sepsis, circulatory failure, or other acute illnesses is a frequent setting [1,4]. Severe lactic acidosis may also occur after massive intentional or accidental ingestion, whereas profound MALA is uncommon during stable therapeutic use when drug elimination is preserved [1]. Importantly, serum creatinine measured at presentation may not fully reflect renal function during the preceding period in which metformin accumulation occurred.

Management of severe MALA can be challenging because treatment requires correction of precipitating physiologic abnormalities while determining when extracorporeal treatment is warranted. Extracorporeal therapy may be required in patients with severe biochemical or clinical toxicity to facilitate metformin removal and correction of profound acidemia [5].

We report the case of a patient receiving metformin who developed profound high-anion-gap metabolic acidosis with a peak lactate concentration of 25.5 mmol/L despite a relatively modest serum creatinine at presentation and evidence of recent renal dysfunction. The case is notable for a substantial decline in lactate before initiation of extracorporeal therapy, partial correction of acidemia during supportive treatment including intravenous sodium bicarbonate, and sequential treatment with high-efficiency intermittent hemodialysis (iHD) followed by continuous venovenous hemodialysis (CVVHD) as a planned strategy to provide rapid initial treatment followed by sustained extracorporeal therapy toward biochemical targets. Hemodynamic intolerance developed during iHD and further favored the transition to continuous therapy. The precisely time-aligned biochemical and treatment course provides an opportunity to examine the indications for extracorporeal treatment in suspected MALA and the limitations of attributing lactate clearance solely to dialysis.

## Case presentation

A 47-year-old man with type 2 diabetes mellitus treated with metformin, cardiomyopathy/heart failure, coronary artery disease status post percutaneous coronary intervention, schizophrenia (treated), and a history of polysubstance use presented on Day 1 with progressive somnolence and altered mental status. On arrival, he was profoundly hypoglycemic, with a glucose level of approximately 39 mg/dL, hypothermic at approximately 95.6°F, and markedly tachypneic, with a respiratory rate of approximately 39 breaths/minute. He was initially hypertensive, with a blood pressure of approximately 180/120 mmHg, rather than hypotensive. Because of worsening encephalopathy and inability to protect his airway, he subsequently required endotracheal intubation and mechanical ventilation.

The dominant initial laboratory abnormality was severe high-anion-gap metabolic acidosis. Serum total carbon dioxide was <5 mmol/L, with an anion gap of approximately 30 mEq/L. Serum lactate was 24.1 mmol/L shortly after presentation and peaked at 25.5 mmol/L. The earliest reported arterial pH was approximately 6.97-7.02; subsequent timed arterial blood gases demonstrated a pH of 7.15 at 14:28 and 7.11 at 16:43 on Day 1. Despite the severity of the metabolic disturbance, serum creatinine was 1.18 mg/dL on admission and increased to 1.49 mg/dL later that day, with only modest azotemia (Table 1).

**Table 1:** Key findings at presentation ~ indicates an approximate value.

Variable	Result	Reference range
Glucose	~39 mg/dL	70–99 mg/dL
Lactate, initial	24.1 mmol/L	≤2.0 mmol/L
Peak lactate	25.5 mmol/L	≤2.0 mmol/L
Serum total CO₂	<5 mmol/L	22–29 mmol/L
Anion gap	~30 mEq/L	6–16 mEq/L
Earliest reported arterial pH	~6.97–7.02	7.35–7.45
Creatinine	1.18 mg/dL	0.71–1.16 mg/dL
Creatinine, Day 1 at 13:21	1.49 mg/dL	0.71–1.16 mg/dL
β-hydroxybutyrate	~0.3 mmol/L	<0.50 mmol/L
Measured serum osmolality	~316 mOsm/kg	275–295 mOsm/kg
Calculated serum osmolality	~296–297 mOsm/kg	275–295 mOsm/kg
Osmolal gap	~19–20 mOsm/kg	Not applicable; calculated from measured and calculated osmolality

Review of the preceding renal course showed recent acute kidney injury. Serum creatinine had increased from 1.36 mg/dL 21 days before Day 1 to a peak of 2.22 mg/dL 14 days before Day 1, followed by gradual improvement to 1.97 mg/dL 12 days before Day 1 and 1.18 mg/dL at the current presentation. Subsequent recovery to 0.88-1.07 mg/dL suggested an apparent baseline of approximately 0.9-1.1 mg/dL and favored recent AKI rather than established stage 3 chronic kidney disease. This history raised the possibility that metformin accumulation may have occurred during the preceding period of impaired renal clearance despite the relatively preserved creatinine measured at the time of the acute presentation.

Because of the combination of severe high-anion-gap metabolic acidosis and an osmolal gap of approximately 20 mOsm/kg, toxic alcohol ingestion was considered. Toxicologic testing did not identify a clear alternative explanation for the metabolic derangement. Ethylene glycol was <10 mg/dL, ethanol was <10 mg/dL, salicylate was <5 mg/dL, and the tricyclic antidepressant screen was negative. β-hydroxybutyrate was only approximately 0.3 mmol/L, making ketoacidosis an unlikely major contributor. Urine toxicology was positive for amphetamine/methamphetamine, while testing for cocaine, opiates, fentanyl, methadone, phencyclidine, cannabinoids, benzodiazepines, and barbiturates was negative (Table 2).

**Table 2:** Toxicologic and metabolic evaluation ~ indicates an approximate value.

Investigation	Result	Reference range/cutoff	Interpretation
Ethylene glycol	<10 mg/dL	<10 mg/dL	No significant level detected
Ethanol	<10 mg/dL	<10 mg/dL	Negative
Salicylate	<5 mg/dL	15–30 mg/dL	Below therapeutic/reference interval; no significant salicylate exposure
Tricyclic antidepressant screen	<40 ng/mL	80–200 ng/mL	Negative
Acetaminophen	11.2 mg/L, then <3 mg/L	<3 mg/L	Low-level exposure
β-hydroxybutyrate	~0.3 mmol/L	<0.50 mmol/L	No significant ketoacidosis
Amphetamine/methamphetamine screen	Positive	Negative; cutoff <1000 ng/mL	Possible contributing stimulant exposure
Cocaine metabolite	Negative	Negative; cutoff <300 ng/mL	Negative
Fentanyl	Negative	Negative; cutoff <1.5 ng/mL	Negative
Opiates	Negative	Negative; cutoff <300 ng/mL	Negative
Methadone	Negative	Negative; cutoff <300 ng/mL	Negative
PCP (phencyclidine)	Negative	Negative; cutoff <25 ng/mL	Negative

The overall biochemical pattern was therefore most consistent with severe lactic acidosis. Given the patient's metformin exposure, recent impairment in kidney function, profound lactate elevation, and severe acidemia, metformin-associated lactic acidosis was considered the leading diagnosis, although the process was considered potentially multifactorial because of concurrent critical illness and stimulant exposure.

Initial management included discontinuation of metformin, correction of hypoglycemia with intravenous dextrose, airway protection, hemodynamic and respiratory support, and repeated intravenous sodium bicarbonate administration for severe acidemia. This included two 50-mEq intravenous boluses and an infusion containing 150 mEq of sodium bicarbonate in 5% dextrose at 100 mL/hour. The last confirmed sodium bicarbonate dose before dialysis was administered at 15:07 on Day 1. During this period, the lactate concentration had already begun to decline, falling from a peak of 25.5 mmol/L at 11:51 to 20.8 mmol/L at 16:43 and 16.0 mmol/L at 19:30 on Day 1. Arterial pH simultaneously improved from 7.11 at 16:43 to 7.30 at 19:54, before initiation of extracorporeal therapy. Because sodium bicarbonate had been administered repeatedly, the pre-dialysis improvement in acidemia was interpreted as occurring during active supportive treatment rather than as spontaneous correction.

Despite this partial improvement, the initial severity of the metabolic disturbance, including a peak lactate concentration of 25.5 mmol/L, profound acidemia, and depressed level of consciousness, prompted initiation of extracorporeal therapy. A single session of high-efficiency intermittent hemodialysis was started at 20:12 on Day 1. The prescribed treatment duration was four hours, with a blood flow rate of 400 mL/min and a dialysate flow rate of 800 mL/min. The session continued until 23:28 on Day 1, for an actual duration of 3 hours and 16 minutes. Because pulmonary edema was also present, ultrafiltration was performed with a prescribed net ultrafiltration goal of 1.5 L. During iHD, lactate decreased to 9.3 mmol/L at 21:41 and 8.4 mmol/L at 22:15. The treatment strategy was to use iHD for rapid initial extracorporeal therapy and then transition to CVVHD to continue treatment toward biochemical targets rather than prolong the intermittent session. During iHD, the patient developed hypotension and briefly required norepinephrine support, which further favored the planned transition to continuous therapy.

CVVHD was initiated at 23:34 on Day 1, six minutes after discontinuation of iHD, and continued until 09:22 on Day 2. CVVHD was prescribed at approximately 35 mL/kg/hour. Lactate was 5.7 mmol/L at 23:33 on Day 1, immediately after iHD and before CVVHD initiation. During CVVHD, lactate decreased further to 4.7 mmol/L at 00:15, 4.1 mmol/L at 03:46, 3.1 mmol/L at 05:53, 2.4 mmol/L at 07:54, and 2.3 mmol/L at 08:35 on Day 2. Twenty minutes after CVVHD discontinuation, lactate was 1.8 mmol/L and remained normal thereafter without significant rebound (Table 3).

**Table 3:** Lactate trajectory in relation to extracorporeal therapy RRT, renal replacement therapy; iHD, intermittent hemodialysis; CVVHD, continuous venovenous hemodialysis.

Day/time	Lactate (mmol/L)	Treatment phase
Day 1, 08:30	24.1	Pre-RRT
Day 1, 09:05	24.0	Pre-RRT
Day 1, 11:51	25.5	Pre-RRT, peak
Day 1, 16:43	20.8	Pre-RRT
Day 1, 19:30	16.0	Pre-RRT
Day 1, 20:12	—	iHD started
Day 1, 21:41	9.3	iHD
Day 1, 22:15	8.4	iHD
Day 1, 23:28	—	iHD stopped
Day 1, 23:33	5.7	Transition
Day 1, 23:34	—	CVVHD started
Day 2, 00:15	4.7	CVVHD
Day 2, 03:46	4.1	CVVHD
Day 2, 05:53	3.1	CVVHD
Day 2, 07:54	2.4	CVVHD
Day 2, 08:35	2.3	CVVHD
Day 2, 09:22	—	CVVHD stopped
Day 2, 09:42	1.8	Post-RRT
Day 2, 13:28	1.6	Post-RRT
Day 2, 22:34	1.5	Post-RRT
Day 3, 02:05	1.1	Post-RRT

Acid-base parameters corrected rapidly during the same period. Serum total CO₂ increased from 6 mmol/L at 13:21 on Day 1 to 23 mmol/L at 23:33, although no chemistry measurement was available between 13:21 and the initiation of iHD; therefore, this increase could not be attributed separately to intravenous bicarbonate versus dialysis. Arterial pH increased to 7.61 at 00:15 on Day 2 and remained markedly alkalemic, reaching 7.65 at 05:53 before gradually returning toward the normal range. The degree of alkalemia was greater than could be explained by the measured serum bicarbonate alone, suggesting an additional respiratory alkalosis component during mechanical ventilation.

Kidney function initially appeared to improve during extracorporeal therapy, with creatinine decreasing to approximately 0.96-0.98 mg/dL; however, this immediate reduction was interpreted as an effect of extracorporeal creatinine clearance rather than evidence of instantaneous renal recovery. After RRT was discontinued, creatinine rebounded to 1.96 mg/dL by the evening of Day 2 and remained near 1.9 mg/dL early on Day 3. It subsequently declined spontaneously without further dialysis to 1.60 mg/dL on Day 4, 1.07 mg/dL on Day 5, and 0.88 mg/dL later that day. Native urine output also increased progressively during recovery.

No additional extracorporeal therapy was required. Lactate remained normal after CVVHD discontinuation, metabolic acidosis did not recur, and renal function returned to the apparent pre-illness baseline. The temporal relationships among intravenous sodium bicarbonate administration, extracorporeal therapy, lactate, arterial pH, serum total CO₂, creatinine, and urine output are shown in Figure 1.

**Figure 1:**
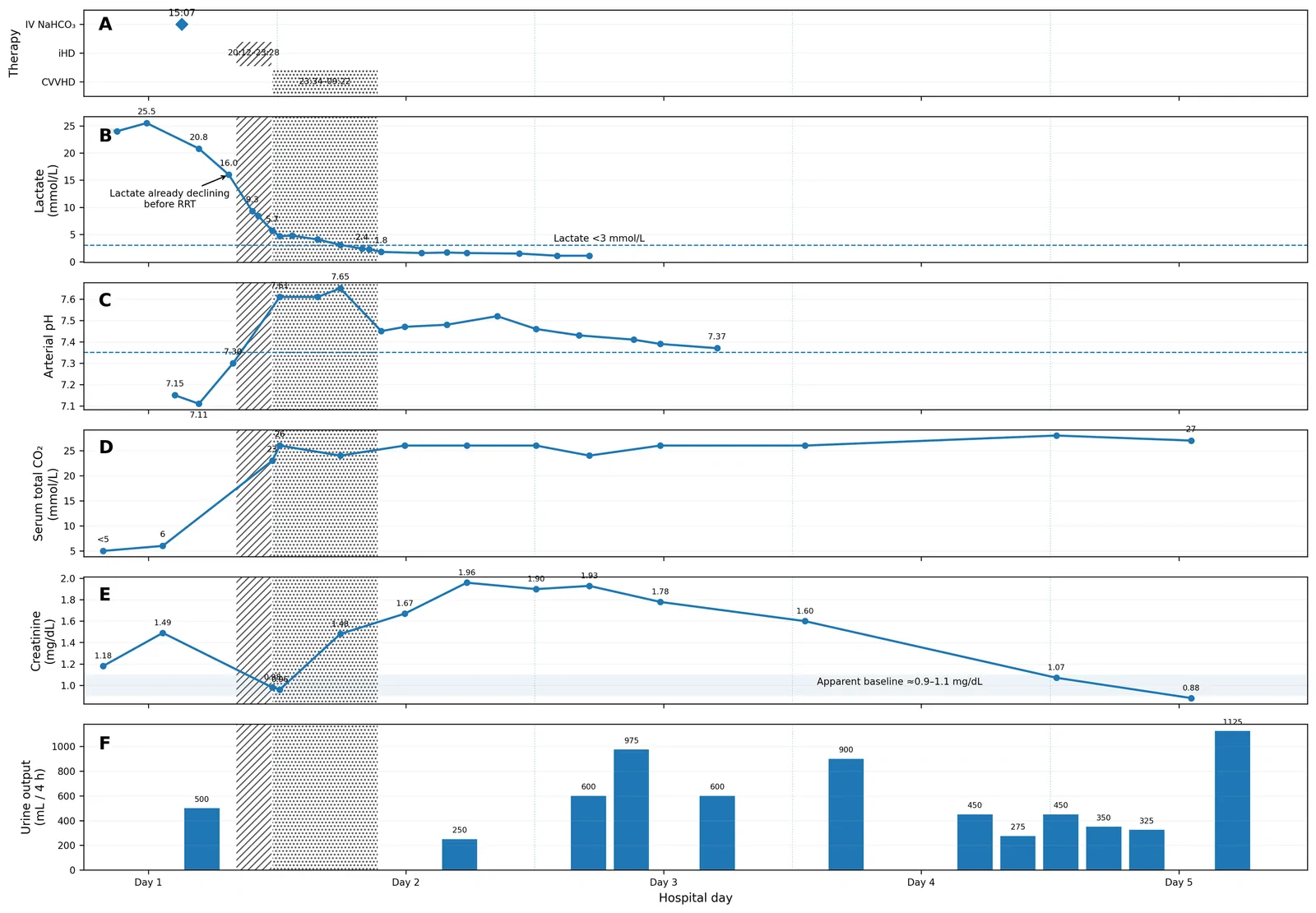
Time-aligned metabolic and renal course relative to supportive therapy and renal replacement therapy (A) Timing of the last confirmed pre-iHD intravenous sodium bicarbonate administration, iHD, and CVVHD. (B) Serial serum lactate concentrations; the dashed horizontal line marks the EXTRIP discontinuation target of lactate <3 mmol/L. (C) Serial arterial pH; the dashed horizontal line marks pH 7.35. (D) Serial serum total CO₂ concentrations. (E) Serial serum creatinine concentrations; the shaded band indicates the apparent baseline creatinine range of approximately 0.9–1.1 mg/dL. (F) Urine output recorded in 4-hour intervals. Values are plotted according to their actual measurement times from Day 1 through Day 5. The shaded treatment periods correspond to iHD and CVVHD and are aligned across panels. Laboratory values were not interpolated. The serum total CO₂ value reported as <5 mmol/L was plotted at 5 mmol/L for graphical display while retaining its designation as <5 mmol/L. The figure was generated in Visual Studio Code using Python 3.13.7 with the pandas and Matplotlib libraries from time-stamped clinical data. EXTRIP: Extracorporeal Treatments in Poisoning; iHD: intermittent hemodialysis; CVVHD: continuous venovenous hemodialysis; CO₂: carbon dioxide; RRT: renal replacement therapy; NaHCO₃: sodium bicarbonate.

The patient was extubated and had a stable course. He reported taking acetaminophen daily for several months for joint pain but denied intentional or accidental overdose of metformin. He was discharged with insulin to manage his diabetes and instructions to avoid further metformin use and limit the use of acetaminophen. He expressed good insight and was advised to follow up with his primary care physician for ongoing management.

## Discussion

Metformin-associated lactic acidosis is uncommon but potentially fatal and typically develops when metformin accumulation occurs in conjunction with another physiologic insult. Metformin is eliminated unchanged by the kidneys; therefore, acute reductions in renal clearance caused by volume depletion, sepsis, hemodynamic compromise, or acute kidney injury can substantially increase drug exposure. Importantly, MALA is often not a purely drug-induced phenomenon. Critically ill patients may simultaneously have increased lactate production from tissue hypoperfusion, catecholamine excess, or other systemic illness and impaired lactate utilization related to metformin. For this reason, the term metformin-associated lactic acidosis is appropriate when metformin is implicated, but other contributors to lactic acidosis are also plausible [1].

In the present case, several features supported MALA, including ongoing metformin exposure, recent AKI, profound high-anion-gap metabolic acidosis, lactate exceeding 25 mmol/L, and altered consciousness. The relatively preserved creatinine at presentation does not exclude preceding metformin accumulation. Serum creatinine had reached 2.22 mg/dL approximately two weeks earlier and subsequently improved to 1.18 mg/dL by the time of presentation. Thus, metformin exposure during the preceding period of impaired clearance may have been more relevant than the single creatinine measured on arrival. Nevertheless, the positive urine amphetamine/methamphetamine screen raised the possibility of stimulant exposure. Together with critical illness and potential hemodynamic contributors, this supported a potentially multifactorial process. In the absence of a measured serum metformin concentration, the case is therefore best considered suspected MALA rather than proven isolated metformin toxicity.

The severity of the metabolic disturbance rather than the degree of azotemia was the principal indication for extracorporeal treatment. The Extracorporeal Treatments in Poisoning (EXTRIP) Workgroup recommends extracorporeal treatment for severe metformin poisoning when lactate is >20 mmol/L, arterial pH is ≤7.0, or supportive measures fail. Treatment is suggested at lactate concentrations of 15-20 mmol/L or pH of 7.0-7.1, while shock, impaired kidney function, hepatic failure, and decreased consciousness lower the threshold for intervention [5]. Bicarbonate-buffered intermittent hemodialysis is the preferred initial modality because it provides rapid metformin clearance and correction of severe acidemia, while continuous kidney replacement therapy may be used when intermittent treatment is unavailable or poorly tolerated [5].

This patient had a peak lactate of 25.5 mmol/L, profound acidemia, and markedly depressed consciousness requiring intubation and therefore fulfilled accepted criteria for extracorporeal treatment despite a serum creatinine of only 1.18-1.49 mg/dL. Dialysis in severe MALA should not be viewed solely as renal replacement for advanced kidney failure. Metformin has a low molecular weight and minimal protein binding and is amenable to extracorporeal removal, while bicarbonate-buffered dialysis simultaneously provides rapid acid-base correction [5].

High-efficiency intermittent hemodialysis was appropriately initiated to provide rapid initial extracorporeal clearance and acid-base correction. The treatment strategy was sequential: transition to CVVHD was planned after the initial high-efficiency phase to provide sustained extracorporeal therapy until biochemical targets were achieved, rather than relying on a prolonged intermittent treatment alone. During iHD, the patient developed hypotension requiring brief norepinephrine support, which further favored transition to continuous therapy. Intermittent hemodialysis provides high instantaneous metformin clearance, whereas continuous therapy generally provides slower but sustained extracorporeal clearance and may be better tolerated in hemodynamically unstable patients [5-7]. The sequential strategy used in this case therefore combined rapid initial clearance with continued extracorporeal therapy toward treatment targets; hemodynamic intolerance reinforced, rather than solely determined, the transition to CVVHD.

An important observation is that the serum lactate had already begun to decline before extracorporeal therapy. Lactate peaked at 25.5 mmol/L at 11:51 and declined to 20.8 mmol/L at 16:43 and 16.0 mmol/L at 19:30, while iHD did not begin until 20:12. Thus, approximately 37% of the decline from the peak lactate concentration had occurred before dialysis.

This temporal relationship argues against attributing the entire reduction in lactate to extracorporeal removal. Serum lactate reflects the balance between ongoing production and endogenous metabolism, predominantly by the liver with an additional contribution from the kidneys. Once production decreases and endogenous metabolism recovers, circulating concentrations may decline substantially without extracorporeal removal. In critically ill patients receiving continuous venovenous hemofiltration with dialysis, filter lactate clearance represented less than 3% of total plasma lactate clearance in a prospective physiologic study [8]. Similarly, high-volume continuous hemofiltration has been shown to provide lactate clearance that is small relative to the rate of lactate production in severe critical illness [9].

The principal therapeutic rationale for extracorporeal treatment in severe MALA should therefore be understood as metformin removal and correction of severe acid-base abnormalities, rather than simply mechanical removal of circulating lactate [5,8,9]. In this patient, improvement in the underlying balance between lactate production and endogenous clearance had clearly begun before dialysis, while rapid metabolic correction continued during iHD and CVVHD.

The acid-base trajectory should also be interpreted separately from the lactate trajectory. The patient received multiple doses of intravenous sodium bicarbonate before RRT, with the last confirmed pre-dialysis administration at 15:07. Arterial pH subsequently improved from 7.11 at 16:43 to 7.30 at 19:54, before iHD began. Therefore, this improvement should not be considered spontaneous correction of acidemia. Intravenous bicarbonate increases extracellular bicarbonate concentration and can raise arterial pH but does not remove metformin or directly correct the underlying cause of lactate accumulation [5,10]. Consequently, the pre-RRT improvement in pH can reasonably be attributed in part to supportive alkali therapy, whereas the simultaneous fall in lactate indicates improvement in the balance between lactate production and endogenous metabolism.

Interpretation of the bicarbonate response is limited by the timing of chemistry measurements. The next available serum total CO₂ after the pre-RRT value of 6 mmol/L at 13:21 was 23 mmol/L at 23:33, immediately after completion of iHD. Because no chemistry CO₂ measurement was obtained between bicarbonate administration and initiation of dialysis, the increase from 6 to 23 mmol/L cannot be quantitatively partitioned between intravenous bicarbonate, recovery of the underlying metabolic process, and bicarbonate-buffered hemodialysis.

EXTRIP recommends discontinuing extracorporeal treatment when both lactate is <3 mmol/L and pH is >7.35, followed by close monitoring because recurrent metabolic abnormalities may require additional treatment [5]. In this patient, lactate reached 2.4 mmol/L at 07:54 and 2.3 mmol/L at 08:35, while pH remained >7.35. CVVHD was discontinued at 09:22. Twenty minutes later, lactate was 1.8 mmol/L and remained approximately 1.1-1.7 mmol/L thereafter without recurrent acidosis. Thus, the biochemical criteria for discontinuation were satisfied, and there was no clinically significant rebound requiring additional dialysis.

The serum creatinine trajectory also requires careful interpretation because creatinine itself is removed by dialysis. The fall to approximately 0.96-0.98 mg/dL during extracorporeal treatment should therefore not be interpreted as immediate intrinsic renal recovery. Following discontinuation of RRT, creatinine increased to approximately 1.9-2.0 mg/dL. More convincing evidence of native renal recovery was the subsequent spontaneous decline without further dialysis to 1.60 mg/dL, 1.07 mg/dL, and ultimately 0.88 mg/dL, accompanied by recovery of urine output.

Overall, this case illustrates several clinically relevant features of severe suspected MALA. Profound lactic acidosis may be present despite relatively preserved serum creatinine at the time of presentation, particularly when renal function was impaired during the preceding period. Extracorporeal therapy may be indicated for toxicologic and acid-base reasons rather than azotemia alone. Finally, biochemical improvement may begin before extracorporeal treatment, making careful temporal alignment of lactate, acid-base parameters, bicarbonate administration, and RRT essential to avoid over-attributing metabolic recovery to dialysis.

## Conclusions

This case demonstrates that severe suspected MALA can occur despite relatively preserved serum creatinine at presentation, particularly when renal function has recently been impaired. The decision to initiate extracorporeal treatment should therefore be based on the severity of the acid-base disturbance, lactate concentration, neurologic status, hemodynamic condition, and overall clinical context rather than the degree of azotemia alone. In this patient, high-efficiency intermittent hemodialysis was used initially for rapid extracorporeal treatment, with planned transition to CVVHD for sustained therapy toward biochemical targets. Hemodynamic intolerance during iHD further supported the transition but was not its sole rationale. The sequential use of iHD followed by CVVHD reflected the clinical circumstances of this individual case and should not be interpreted as a generally applicable treatment sequence.

The case also highlights the importance of interpreting biochemical changes according to the timing of treatment. Lactate had already begun to decline before dialysis, whereas partial correction of acidemia occurred during supportive treatment that included repeated intravenous sodium bicarbonate. Extracorporeal therapy was undertaken to facilitate metformin removal and correction of severe acidemia; however, without serum metformin concentrations or clearance measurements, the specific contribution of metformin removal cannot be quantified. Time-aligning laboratory trends with therapeutic interventions can therefore provide a more accurate assessment of the contribution of extracorporeal therapy in severe MALA.
